# The NGS WikiBook: a dynamic collaborative online training effort with long-term sustainability

**DOI:** 10.1093/bib/bbt045

**Published:** 2013-06-21

**Authors:** Jing-Woei Li, Dan Bolser, Magnus Manske, Federico Manuel Giorgi, Nikolay Vyahhi, Björn Usadel, Bernardo J. Clavijo, Ting-Fung Chan, Nathalie Wong, Daniel Zerbino, Maria Victoria Schneider

**Keywords:** next-generation sequencing, bioinformatics, training, collaborative learning, best practice

## Abstract

Next-generation sequencing (NGS) is increasingly being adopted as the backbone of biomedical research. With the commercialization of various affordable desktop sequencers, NGS will be reached by increasing numbers of cellular and molecular biologists, necessitating community consensus on bioinformatics protocols to tackle the exponential increase in quantity of sequence data. The current resources for NGS informatics are extremely fragmented. Finding a centralized synthesis is difficult. A multitude of tools exist for NGS data analysis; however, none of these satisfies all possible uses and needs. This gap in functionality could be filled by integrating different methods in customized pipelines, an approach helped by the open-source nature of many NGS programmes. Drawing from community spirit and with the use of the Wikipedia framework, we have initiated a collaborative NGS resource: The NGS WikiBook. We have collected a sufficient amount of text to incentivize a broader community to contribute to it. Users can search, browse, edit and create new content, so as to facilitate self-learning and feedback to the community. The overall structure and style for this dynamic material is designed for the bench biologists and non-bioinformaticians. The flexibility of online material allows the readers to ignore details in a first read, yet have immediate access to the information they need. Each chapter comes with practical exercises so readers may familiarize themselves with each step. The NGS WikiBook aims to create a collective laboratory book and protocol that explains the key concepts and describes best practices in this fast-evolving field.

## INTRODUCTION

As Anthony Hey from Microsoft Research wrote in his inspiring book ‘The Fourth Paradigm: Data Intensive Scientific Discovery’, we are transforming into another fundamental way of scientific endeavour: an era of data exploration. Typically, big data sets are generated or simulated, and then analysed by advanced algorithms. Next-generation sequencing (NGS) is one of the major contributors responsible for the data deluge in science. It has become an essential technology in much of biomedical research [1–4]. NGS comprises not only a number of substantially different technologies but also of different applications (e.g. whole genome, exome and transcriptome sequencing). The available tools are many, with, at times, overlapping and complementary functionality [5–8]; helped by the open-source nature of many of these, custom combinations of different tools in *ad hoc* pipelines is a common practice in NGS data analysis. However, despite the wide range of interested users (from computational scientists to life scientists with little computing experience), tutorials are few and sparse, and seldom offer the desired combination of functionality.

Training users in NGS bioinformatics analysis is challenging not only because of its interdisciplinary nature, but also because of the heterogeneity of trainee backgrounds and the extensive technological changes that are continually happening in the field [9]. As we are going to discuss, extensive sharing of experience is imperative for sustainable training, which is made possible by online collaborative efforts.

## PHILOSOPHY BEHIND THE NGS WIKIBOOK

### Nine simple rules to begin with NGS analysis

Bioinformatics has established itself as a reliable partner of experimental biology. Unfortunately, various myths that were raised 12 years ago still prevail today [10]. These disparate expectations and perceptions due to the different mindsets possessed by life scientists and bioinformaticians need to be reconciled (Table 1). Although the exact NGS analysis methods tend to evolve over time, we would like to suggest nine simple rules for novices who are about to engage in bioinformatics.

**Table 1:** Examples of disparate expectations of NGS bioinformatics by life scientists and bioinformaticians

Misconception on bioinformatics	The reality
It is a rapid and easy publishing field.	The rather rapid publishing in the bioinformatics field is possible because it has sped up the observation and allowed analysis at an unprecedented speed compared with traditional experimental approaches.
A final result is generated automatically by pressing a button.	There exists no magic programme to do everything. Setting up computationally is an expensive operation and has to be done with great attention to details and understanding of the procedures. Besides, every result should be replicable by repeating the analysis with a slight change in parameters or with a different fundamental approach.
NGS analysis is all about alignment/read mapping that anyone can master within one day.	Choosing the optimal approach depends on the biological question being asked and the NGS technology used.

## RULE 1: DO NOT FEAR THE COMMAND LINE

Most academic software runs in command line interface. The initial learning curve of the command line may be steep for a wet-lab biologist. But once mastered, command line interface empowers users with simple yet powerful commands. Users should familiarize themselves with shell commands and scripting. Many repetitive tasks can be automated. Alternatively, open-source (e.g. GeneProf [11]) and commercial (e.g. CLC bio, Partek) software often offers user-friendly, one-step analysis platforms. Because of the wide spread of kits in biology, in which difficult procedures are standardized into kit forms, these commercial software solutions are especially tempting for wet-lab experimentalists because they are accustomed to these packages. However, most NGS analyses are still not mature enough to be implemented into a single tool. And for non-standardized analyses, where most novel discoveries are found, it is essential to be equipped with at least one programming or scripting language. In a nutshell, learning programming makes a biologist more efficient in data analysis.

## RULE 2: KNOW THE CONVENTIONS

The file format confusion represents one of the biggest challenges faced by bioinformaticians [12]. Before starting NGS data analysis, it is necessary to understand the various file formats that are commonly used. These include the FASTQ format and its various quality encoding systems [13], the SAM format for short read alignment over a reference genome/transcriptome [14], the standard genetic variant call format (VCF) [15] and the differences in genome coordinate systems used in major databases and annotation files [16].

## RULE 3: READ INTRODUCTORY REVIEWS

A thorough understanding of NGS terms is a prerequisite for data analysis. The BlueSEQ Knowledge Bank, for instance, maintains an updated technologies perspective, a glossary list of NGS terms and a list of NGS-related blogs. The journal *Nature* provides a list of introductory reviews [17]. These resources provide a bird’s-eye view of the field and the various concepts behind the analysis strategies.

## RULE 4: START WITH QUALITY CHECKING

Always start with understanding the sequence data. There are various metrics to assess the quality of the data, including, but not limited, to the following: (i) sequence quality: no drastic drop of average base calling accuracy should occur throughout the reads; (ii) per base sequence content: in general, assuming the data are a random sample from the sequence space, then at each position the contribution of base identity should be identical. For details, refer to the NGS WikiBook. Often, strange bugs pop up because the sequence data do not fit the tools’ requirement. Some common features in the file being processed that may fail aligners include truncated sequence files, variable read lengths not supported by the tool or incorrect read name format. In most cases, these errors could be avoided by carefully scrutinizing the sequence data and the tools’ documentation before a considerable time is spent on analysis.

## RULE 5: PLAN FOR MISTAKES AND DOCUMENT WORKFLOW

Trial and error is the greatest ally of bioinformaticians. Always prepare to make mistakes during preliminary analysis. To identify potential bugs in the pipeline, always test the proposed workflow in a tiny data set first, before applying it into the real data sets. Besides, data analysis is an iterative process that leads to multiple possible solutions. Especially, when many tools are combined, it may be hard to reconstruct the steps that led to a specific result by other members of the scientific community or even by the same researcher. Therefore, it is important to protocol every part of the analysis process and store all data that were used to generate the final result.

## RULE 6: ALWAYS GET INFORMED AND GET HELP IF STUCK

NGS is a rapidly evolving field. Novel analysis techniques and tools appear every day. To keep current of developments in the field, regularly visit online resources, such as GenomeWeb and Bio-IT World, and follow hashtag #NGS, #genomics and #sequencing on Twitter. Obtaining help from online communities, including Biostars [18] and SEQanswers [19], is a norm in the NGS field. When receiving help from online scientific communities, follow the guidelines framed by Dall’Olio *et al.* [20].

## RULE 7: USE AN EFFICIENT INTEGRATIVE APPROACH

Galaxy [21] and GenePattern [22] are open-source integrative platforms that are particularly attractive to biochemical experimentalists, as they allow bioinformatics novices to carry out computationally intensive analyses online (in either a public server or private cloud). Numerous tools encompassing read mapping, variant discovery, the legendary tuxedo pipeline for differential expression analysis [23] and visualization methods are available. These platforms also ensure transparency and reproducibility by recording metadata from every analysis, including the tools, versions and parameter settings used during the analysis. Nevertheless, these platforms do not automate the entire analysis procedure and can be useful only if aware of what assumptions (e.g. the rationales behind the parameter choice) are being made. Note that these platforms may not necessarily be the best choice for many of the questions being addressed. Besides, many NGS tools are still actively being developed, and therefore the tools maintained in the integrative platforms may not be updated. Users should resort to the original release sites if bleeding edge functions of respective tools are needed.

## RULE 8: AVOID REINVENTING THE WHEEL

In bioinformatics, solutions to some common tasks and problems often have been codified and made open-source. A 15-min Google search often saves 2–3 days in implementing the codes from scratch. For example, Biopython and BioPerl are open-source frameworks that have implementation for common results parsing and manipulation [24]. But, of course, if users find a task that strangely has not been solved, they should share their 2–3 days’ script with the community to help the future bioinformatics traveler.

## RULE 9: EDUCATION IS IMPORTANT

Bioinformatics is a broad subject; one must expect to learn along the way. As users navigate through NGS analysis, they will soon find no single tool can deal with all the tasks they want to accomplish. As novel algorithms are continuously developed for discovery in NGS analysis (e.g. driver mutation in cancer [25]), it is important to keep on trend of the tools’ development. Although the NGS WikiBook focuses on guiding users to optimally use bioinformatics tools, knowing merely how to run the tools is insufficient. Theoretical courses with broader coverage and knowledge on fundamental concepts are also important. Some highly interactive university-level courses are described elsewhere [26, 27].

## THE NGS WIKIBOOK

Standardization is the key towards reusability of sequence data and reproducibility of the data analysis. After years of effort, the data format of NGS has matured. The current effort on the adoption of a data-sharing standard, such as the Minimum Information about a high-throughput SeQuencing Experiment (MINSEQE), aims to provide guidelines on submission of sequence data to the public repository [28]. Such guidelines facilitate the reuse of existing sequence data and ensure adequate information of data would be accessible by other researchers. The adoption of a community-accepted analysis strategy is essential towards analysis reproducibility. Built on the philosophy summarized in the aforementioned nine simple rules, the NGS WikiBook represents one of the ways to consolidate the NGS training efforts. This set of materials is designed for wet-lab biologists and bioinformatics novices who demonstrate interest in NGS data analysis and need conceptual overview and practical guidelines. Experts in the community could contribute more advanced materials as the needs and trends in the field develop. The flexibility of online material allows readers to ignore details in a first read, yet have immediate access to the details they need. The overall structure and style is in priority designed for the non-bioinformatician reader (Table 2).

**Table 2:** Summary of content in the NGS WikiBook

Chapter	Theme	What is it about?
1	Introduction	Overview of the field. Starting with sequencing technologies, their properties, strengths and weaknesses, covering the various biologies that they assay and finishing with a section on common sequencing terminology. An overview of a typical sequencing workflow is presented.
2	Big data	Some of the (perhaps unexpected) difficulties that arise when dealing with typical volumes of NGS data. From shipping hard drives around the world to the amount of computer memory needed to assemble the data when they arrive. File formats, archives and algorithms that have been developed to deal with these problems are discussed.
3	Bioinformatics from the outside	Discussing the interfaces used by bioinformaticians. The command line with its text interface and blinking cursor and also more user-friendly graphical user interfaces (GUIs), which were developed especially for bioinformatics pipelines, are reviewed.
4	Preprocessing	Discussing the best practices of controlling the quality of a NGS data set, and cleaning of low-quality data.
5	Alignment	How to map a set of reads to a reference sequence.
6	DNA variants	How to call variants (single nucleotide variation, copy number variation or structural variations) using mapped reads.
7	RNA	How to determine exons, isoforms and gene expression levels from mapped RNA-seq reads.
8	Epigenetics	Pull-down assays, which are used to determine epigenetic traits such as histone or CpG methylation.
9	Chromatin structure	Technologies used to determine the structure of the chromatin, e.g. the placement of the histones or the physical proximity of different chromosomal regions when the DNA lies in the nucleus.
10	*De novo* assembly	Ways to assemble a genome from NGS reads.
11	*De novo* RNA assembly	Ways to assemble a transcriptome from NGS reads.
12	Authors	Contributors of substantial amount of work to this WikiBook should add themselves to this chapter.

## DISCUSSION

### Importance of training in NGS

Bioinformatics is a field of development and application of computational approaches to acquire, analyse, visualize and archive data generated from biological systems. Since the advent of NGS technologies, life sciences have relied more on quantitative data, the size of which has become larger than ever before. Traditionally, computational scientists lack biology education, whereas biologists know little about computer science. However, to become a competent biologist today, an individual must have many computer skills: knowledge to deal with large amounts of sequence data. Formal bioinformatics education is offered in various countries [29]. Still, some challenges, including the structure and the breadth of the bioinformatics education programme that were identified 10 years ago, are still with us today [30]. Recently, Pevzner *et al.* [31] advocated bioinformatics education through biological question-oriented teaching of computational concepts (e.g. clustering and pattern recognition). Although such an approach pertains to complex computational ideas applied to biological problems, it is of equal importance that biologists are informed of empirical experience so that they can (i) squeeze the maximum amount of information out of their data, and (ii) be able to notice dubious results by better deploying existing tools. These include, but are not limited to, approaches of sequence alignment, variant discovery, expression analysis and *de novo* assembly. Each of these tasks involves specific challenges and is not as straightforward as they may seem. To name a few, accurate discovery of variants and gene fusion require careful choice of mapping tools, fine tuning of parameters and systematic filtering [32].

Practical bioinformatics training is necessary. But then bioinformatics grows in parallel with technology. The training landscape is constantly changing; new tools become available and existing tools are being refined over time. For example, fundamental sequencing technologies that include library preparation methods and sequencing-related algorithms are constantly improving [6]. As of today, more than 70 generic [33] and RNA-Seq [8] short read aligners are available. It is challenging for trainers to keep themselves well informed of the trends of the field as well, as they often come from diverse scientific backgrounds. Besides, much of the material to be covered in lectures and the software to be used may not be a part of their own formal research experience [34].

### Training starts online

For traditional life scientists and bioinformatics novices who want to familiarize themselves with NGS analysis, conducting literature research is an intuitive way. Existing publications with the accompanying data sets have therefore become an important pool of resources for self-learning in the field. Unfortunately, traditional publications often lag significantly behind the state-of-the-art in analysis methods, which evolve rapidly. Besides, the majority of the current articles published in high-impact journals lack sufficient details in the informatics component, making the computational analyses almost irreproducible [35]. Book chapters are invaluable resources for bioinformatics education and provide readers with comprehensive knowledge background on theories and algorithm details. Still, bioinformatics practitioners are often in need of more practical guides [36].

Although workshops remain as an important source for bioinformatics training [37], numerous organizers have started to share their workshop material online for the motivated self-learners worldwide [38, 39]. One of the suggested solutions for bioinformatics training is to provide always up-to-date web-based training materials to allow users to explore a range of current bioinformatics tools and basic algorithms through hands-on exercises [9, 26]. Rosalind [27], for example, is an online platform for learning bioinformatics-related programming skills through problem solving at the students’ own pace and learning common standalone and web-based bioinformatics tools. A majority of bioinformatics could be self-taught through materials scattered around the Internet. Recently, online communities have become important sources of support for increasing numbers of experienced researchers [20]. This coincides with the rise of the two major NGS online communities, namely, BioStar [18] and SEQanswers [19] and its affiliated SEQWiki [40], which aid practical bioinformatics training. BioStar uses a sophisticated platform for asking and answering questions, and answers are rated by the community. SEQanswers, on the other hand, facilitates collective discussion of technologies, methods and semantic information on NGS-related tools. Despite the successes of these online communities, a comprehensive practical guide to the field is still unavailable. Finding central synthesis of NGS know-how is difficult. On the other hand, Wikipedia deposits an encyclopedic description of NGS information but explicitly disallows ‘how-to’ style manuals and instruction. In both platforms, experienced bioinformaticians can navigate through the information by searching for specific keywords, but novices often struggle just to start their first NGS analysis. Therefore, we initiated the NGS WikiBook to provide readers an online training environment, with a focus on ‘how to’. The Wikipedia Foundation is officially encouraging WikiBooks for this type of tutoring approach [41].

## DYNAMIC COLLABORATION AND SUSTAINABILITY

Built on the WikiBooks project hosted by the Wikimedia Foundation, the NGS WikiBook is an open content collection of NGS-related concepts and approaches that leverage the community intelligence (like Wikipedia). Scientists who are familiar with the field are encouraged to edit collaboratively. This group of co-authors reflects this situation; their background ranges from bioinformatics and computer sciences to biology, allowing them to understand the needs of basic NGS training as a user. Some authors are bioinformatics trainers or professors, and all are NGS practitioners.

The sustainability of this community effort depends on input from the entire NGS community. This is encouraged by a low barrier to contribution, one of the foundations of Wikipedia’s own success, which has become an important source of accurate scientific information [42, 43]. Within the NGS WikiBook, many scientific tasks that lack up-to-date published approaches could be tackled and described by a multitude of experts in the field. We welcome contributions and invite anyone involved in NGS to engage with and to contribute to this community effort.

## ONLINE RESOURCES

**The NGS WikiBook:** http://en.wikibooks.org/wiki/Next_Generation_Sequencing_(NGS)

**Bio-IT World: **http://www.bio-itworld.com/

**BioStar:** http://www.biostars.org/

**BlueSEQ Knowledge Bank:** http://blueseq.com/knowledgebank/

**GenomeWeb:** http://www.genomeweb.com/

**NGS aligners Feature comparison:** http://wwwdev.ebi.ac.uk/fg/hts_mappers/

**SEQanswers:** http://seqanswers.com/

**The SEQanswers Wiki:** http://seqanswers.com/wiki/

**Key points**

- There exist plenty of tools for NGS analysis, and the challenge of providing education/training on NGS data analysis is widespread.
- None of these tools satisfies all wishes or needs, but solutions exist for many particular aspects.
- Most of the tools are open-source. By pipeline integration, the deficiencies in functionality of a particular tool may be compensated by the strengths of another.
- The NGS WikiBook provides a ground-up best practice for bioinformatics analysis.

## FUNDING

We are grateful to The Genome Analysis Centre (TGAC) and the Biotechnology and Biological Sciences Research Council (BBSRC) for their support in making this an OA article. J.W. is supported by a General Research Fund (GRF461712) to T.F.C. and a Theme-based Research Scheme (Ref. no. T12–403/11) to N.W., N.V. is supported by an RF 11.G34.31.0068 to S.J.O. and 11.G34.31.0018 to P.P.
